# Cataracte post traumatique rompue négligée

**DOI:** 10.11604/pamj.2022.42.3.33130

**Published:** 2022-05-04

**Authors:** Loubna El Kaissoumi, Basma Mrini

**Affiliations:** 1 Centre Hospitalier Universitaire Ibn Sina, Hôpital des Spécialités, Rabat, Maroc

**Keywords:** Cataracte post traumatique, traumatisme oculaire, contusion, Post traumatic cataract, ocular trauma, contusive trauma

## Abstract

We here report the case of a 26-year-old patient with no particular medical history, who was victim of neglected contusive right eye trauma after physical assault 9 months before his admission. The patient presented to the emergency department with progressive vision loss and leukocoria. Ophthalmological examination performed on his admission showed reduced visual acuity limited to hand move in the traumatized eye. Eye examination using the slit lamp objectified clear cornea, good anterior chamber depth with ruptured cataract in the anterior chamber. In addition, eye tone measured with Goldman tonometer was 15 mmHg. Fundus examination couldn´t be performed and ocular ultrasound didn´t show any vitreous abnormalities or retinal detachment. The examination of the contralateral eye was normal. The patient underwent phacoemulsification with insertion of a posterior chamber lens implant with good outcome.

## Image en médecine

Nous rapportons le cas d´un patient âgé de 26 ans, sans antécédents médicaux particuliers, se disant victime 9 mois avant son admission, d´un traumatisme contusif négligé suite à une agression physique au niveau de son œil droit. Le patient s´est présenté aux urgences pour baisse d´acuité visuelle progressive et leucocorie. L´examen ophtalmologique à l´admission retrouvait une acuité visuelle réduite à « voit la main bouger » au niveau de l´œil traumatisé. A la lampe à fente, la cornée était claire, la chambre antérieure était de bonne profondeur, avec présence d´une cataracte rompue en chambre antérieure. Par ailleurs le tonus oculaire était de 15 mmHg au tonomètre de Goldmann. L´examen du fond d´oeil était inaccessible, et une échographie oculaire réalisée n´a pas objectivé d´anomalies vitréennes ni de décollement de rétine. L´examen de l´œil adelphe était sans anomalies. Le patient a été opéré pour sa cataracte par phacoémulsification et mise en place d´un implant de chambre postérieure, avec bonne évolution.

**Figure 1:**
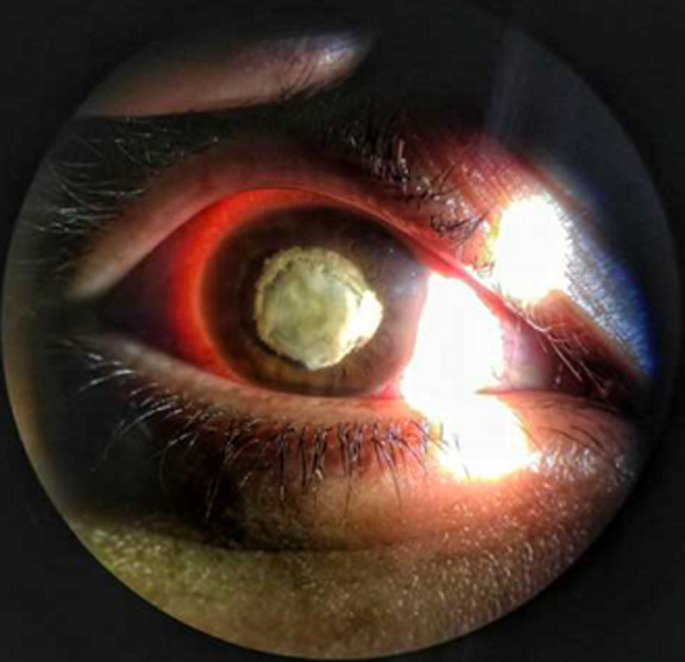
image à la LAF montrant une cataracte post traumatique rompue

